# The three-dimension preclinical models for ferroptosis monitoring

**DOI:** 10.3389/fbioe.2022.1020971

**Published:** 2022-09-23

**Authors:** Yiming Meng, Jing Sun, Guirong Zhang, Tao Yu, Haozhe Piao

**Affiliations:** ^1^ Department of Central Laboratory, Cancer Hospital of China Medical University, Liaoning Cancer Hospital & Institute, Shenyang, China; ^2^ Department of Biobank, Cancer Hospital of China Medical University, Liaoning Cancer Hospital & Institute, Shenyang, China; ^3^ Department of Medical Imaging, Cancer Hospital of China Medical University, Liaoning Cancer Hospital & Institute, Shenyang, China; ^4^ Department of Neurosurgery, Cancer Hospital of China Medical University, Liaoning Cancer Hospital & Institute, Shenyang, China

**Keywords:** ferroptosis, 3D cell culture, anti-tumor therapy, drug, assay

## Abstract

As a new programmed cell death process, ferroptosis has shown great potential and uniqueness in experimental and treatment-resistant cancer models. Currently, the main tools for drug research targeting ferroptosis are tumor cells cultured *in vitro* and tumor models established in rodents. In contrast, increasing evidence indicates that reactivity may differ from modifications in mice or humans in the process of drug screening. With the blossoming of 3D culture technology, tumor organoid culture technology has gradually been utilized. Compared with traditional 2D culture and tumor tissue xenotransplantation, tumor organoids have a significantly higher success rate. They can be cultured quickly and at a lower cost, which is convenient for gene modification and large-scale drug screening. Thus, combining 3D cell culture technology, drug monitoring, and ferroptosis analysis is necessary to develop the impact of ferroptosis-related agents in tumor treatment.

## Introduction

Although modern medicine has made remarkable progress in treating malignant tumors, malignant tumors are still the number one killer of human death. There is still a great demand for developing new anti-tumor drugs (M. Wu et al., 2022). However, most medicines fail to achieve their application from the laboratory to the clinic. Presently, the global screening technology of drug efficacy is mainly based on pharmacodynamic models (Mai et al., 2022). Cell models are one of the drug screening models. In recent years, more additional cell models have been developed for drug screening with the development of cell biology (de Almeida et al., 2022). The effects of the candidate drug on cell activity, apoptosis, migration, differentiation, metabolism, and other behaviors can be examined at the cellular level. The response speed of cell experiments is breakneck, and the efficacy and toxicity of drug candidates can be analyzed in a short period. However, the traditional cell models usually use two-dimensional (2D) cells cultured in a plane whose morphological structure and physiological microenvironment are very different from those in the human body and cannot simulate the *in vivo* situation well (Calpe and Kovacs 2020).

**FIGURE 1:**
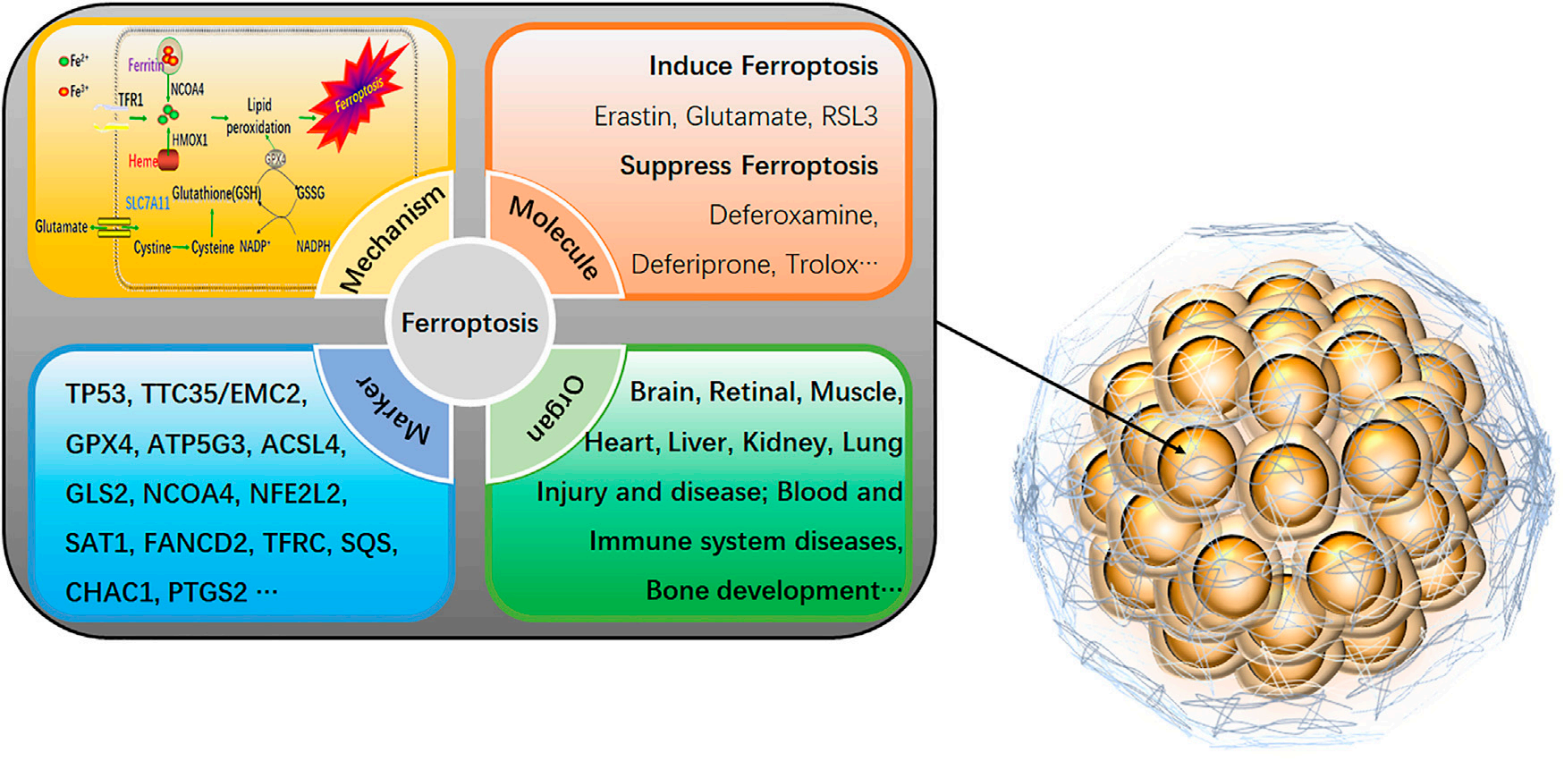
An overview of ferroptosis in 3D cells. The specific mechanism of ferroptosis is as follows: 1) GPX4 inactivation leads to GSH consumption. In cells, GPX4 can convert the peroxy bond of lipid peroxidation into a hydroxyl group and lose its peroxide activity. It is the only glutathione peroxidase used for liposome peroxide reduction. Based on the enzymatic activity of GPX4, its main targets are System Xc-system (responsible for transporting cysteine, a synthetic raw material of GSH into the cell), glutamate-cysteine ligase, glutathione s-transfer enzymes, etc. 2) Direct inactivation of GPX4. In addition to indirectly acting on GSH that activates GPX4, GPX4 inhibitors, squalene synthase, etc., can also directly eliminate GPX4. 3) Iron ion input and iron ion reduction. Input iron ions into cells and ensure that iron ions exist in large quantities in the form of divalent iron ions (Fe^2+^), which can initiate liposome peroxidation through the Fenton reaction. As a new cell death mechanism, ferroptosis has recently been a research hotspot. It can be involved in cancer cell death, neurotoxicity, neurodegenerative diseases, acute renal failure, drug-induced hepatotoxicity, liver and cardiac ischemia/reperfusion injury, T cell immunity, and other life processes (Neurodegeneration, Multi-organ dysfunction, Retinal pigment epithelial cell degeneration, Asthma, Defective kidney repair, Sickle cell disease, etc.). Activating or inhibiting ferroptosis can interfere with the development of diseases. Therefore, it is of great practical significance for the clinical treatment of human diseases to explore the role of ferroptosis in various conditions by sorting out the related genes that affect ferroptosis.

On the other hand, 2D cell models are more sensitive to drug responses due to the lack of intercellular junctions and extracellular matrix, which are prone to provide false-positive data for drug screening. Therefore, more scientific research institutions and drug development companies have begun using three-dimensional (3D) cells as a drug screening model. 3D cells have more similar *in vivo* morphological structure, drug response, gene expression, drug sensitivity, and more robust drug tolerance (Castro et al., 2021). 3D cells have shown great potential as a cell model for next-generation drug screening, which takes into account the advantages of traditional cell models in terms of high throughput, low cost, high efficiency, and animal models closer to the *in vivo* situation. Although drug screening based on 3D cells is still in its infancy, its excellent physiological properties make drug screening based on 3D cell models have broad application prospects (Castro et al., 2021; Pamarthy and Sabaawy 2021). Ferroptosis-related agents are rapidly evolving in developing new drugs and are particularly effective in inhibiting several types of cancer (Fuhrmann and Brune 2022). Ferroptosis is an iron-dependent cell death mode that differs from traditional programmed cell death, such as apoptosis, pyroptosis, and autophagy (Zhang and Liu 2022). It is illustrated by the accumulation of lipid peroxides induced by reactive oxygen species. The main manifestations of ferroptosis are marked mitochondrial shrinkage, increased double-membrane density, reduction or disappearance of mitochondrial cristae but intact cell membrane, standard size of nucleus, and uncondensed chromatin (Stockwell 2022). The core mechanism of ferroptosis is the depletion of the antioxidant peptide glutathione (GSH) in cells by inhibiting the cystine-glutamate anti-transporter, eventually accumulating lethal levels of lipid peroxides (Grube et al., 2022). Unlike programmed cell death, ferroptosis is a regulated cell death that relies on specific molecular mechanisms, so specific drugs and genetic interventions can be exploited to speed up or slow down the ferroptosis process (Figure 1).

Ferroptosis-inducing agents could enhance the antitumor effects of radiotherapy, and ferroptosis inhibitors also alleviate radiotherapy-induced lung injury, pulmonary fibrosis, and myeloid radiation sickness. Therefore, modulating the ferroptosis pathway to obtain the most significant clinical benefit in tumor therapy is a vital issue worthy of further study. (Chin et al., 2022; Peng et al., 2022). In Investigative Dermatology, Wang et al. (S. Wang, et al., 2022) identify the Calcium/Calmodulin Dependent Protein Kinase 2 (CAMKK2)-adenosine monophosphate-activated protein kinase-nuclear factor erythroid2-related factor 2 (NRF2) signaling axis is a negative regulator of ferroptosis and show that inhibition of CAMKK2 improves anti-PD-1 therapy effect. These results provide new opportunities to develop ferroptosis-inducing therapies in combination with immune checkpoint drugs. Recent research supports using 3D culture models to identify therapeutic targets that traditional 2D assays may obscure. They believe that cells’ antioxidant capacity is a new strategy for cancer treatment. Importantly, they observed differences in the sensitivity of inner and outer cells to ferroptosis, suggesting that different tumor regions may have various capacities for oxidative stress and sensitivity to ferroptosis-inducing compounds (Takahashi et al., 2020). Thus, combining 3D cell culture technology, drug monitoring, and ferroptosis analysis is necessary to develop the impact of ferroptosis-related agents in tumor treatment.

### Ferroptosis in the 3D cell culture

Targeting multiple oxidative stress defense programs may be necessary to maximize the killing of all cells in the tumor. So, the therapeutic strategies may be by targeting NRF2 (an oxidative defense transcription factor) (Panieri et al., 2022), directly targeting glutathione peroxidase 4 (GPX4), targeting NRF2 transcriptional targets that affect ferroptosis (e.g., Solute Carrier Family 7 Member 11), or targeting other pathways that affect sensitivity to lipid peroxidation (e.g., Acyl-CoA Synthetase Long-Chain Family Member 4) by pooled CRISPR-Cas9 Screen in 3D culture (Takahashi et al., 2020). For the 3D culture, the cells were plated on Matrigel-coated 96 black well plates in complete media supplemented with 2% Matrigel and 1 μg/ml doxycycline. The spheroid cells were re-fed with fresh media every two or 3 days and fixed with 4% paraformaldehyde at the endpoint. The benefit of the spheroid model allowed us to reveal that high NRF2 activity is required for lung cancer spheroid formation and that loss of NRF2 has dramatic effects on two distinct processes during spheroid formation: proliferation and survival of spheroid cells. They exemplified how NRF2 is a connection between cancer cell architecture and ferroptosis; they notify that NRF2 hyperactivation improves 3D proliferation of non-small cell lung cancer (NSCLC), partly through quelling ferroptosis in the extracellular matrix (ECM)-deprived, inner cells of cancer spheroids. Their study provides a basis for further investigation of the link between the antioxidant response of ferroptosis and the extracellular matrix of the tumor microenvironment (W.L. Wu and Papagiannakopoulos 2020). Developing compounds that target anti-ferroptosis modulators could be a promising approach and particularly effective in tumor combination therapy (Kurt-Celep et al., 2022; Y. Wang, et al., 2022).

### Quantitatively determine ferroptosis in spheroids

In numerous large-scale unexplored drug trials in 2008, the single-layer planar culture model was not enough to predict the actual situation of drugs in tumor tissues, and the experimental results were rarely consistent with clinical trial consequences (Mohr et al., 2022). With the continuous development of cell culture media such as scaffolds, gels, and other matrices and support materials, 3D tumor cell models can simulate *in vitro* a 3D space and microenvironment similar to *in vivo*. This technique has gradually become the most promising cell research model. 3D cultures, including spheroids, are nowadays recognized as a better model of the *in vivo* environment, whereas rare detailed cell death (including ferroptosis) assays are available for 3D cultures (Jung et al., 2022; Yan et al., 2022). Robin Demuynck’s (Demuynck et al., 2020) employed 3D cells culture technology to demonstrate an assay that can effectively identify diverse types of cell death, including ferroptosis, and quantitatively assess cell death in tumor spheroids, the 3D Cell Death Assay (3DELTA). Their method used Sytox dye, intercalated with DNA, as a cell death marker and Triton X-100, which efficiently penetrated all cells in the spheroid, to determine 100% cell death. Spheroids were formed by seeding cells (L929sAhFasmouse fibrosarcoma cells and SK-OV-3-Luc-GFP human ovarian cancer cells) on agarose microwell chips—the chip made by pouring liquid agarose onto a polydimethylsiloxane (PDMS) mold. The cells will form clusters, leading to the formation of spheroids. Sytox dyes intercalate with DNA with a high affinity. Sytox Green is non-luminescent outside viable cells. When cells permeabilize at the end of the cell death process, the plasma membrane is ruptured, and Sytox Green will bind to DNA and fluoresce green. Moreover, it emits green fluorescence, which can then be measured.

The following formula calculated the percentage of the cell death:

$$\frac{{average}_{sytox}\left[ML162\right]-{\;average}_{sytox}\left[background\right]}{{average}_{Sytox}\left[Triton\;X-100\right]-{average}_{sytox}\left[background\right]}\times 100\%$$

Cell ferroptosis induced with 5 μm ML-162. Spheroids stained with Sytox Green (L929) or Sytox Blue (SKOV) and cell death, an increase in fluorescence intensity, was measured 24 h later with a Tecan Spark microplate reader. Afterward, the spheroids permeabilized with Triton X-100 0.05% (v/v). Fluorescence intensity was measured and taken as 100% cell death. After optimization of Sytox concentration, Triton X-100 concentration, and time, it demonstrated that the 3D cell death assay (3DELTA) method could detect the signal of all cells without disintegrating the spheroids. Sytox fluorescence intensities were detected by the Tecan Spark^®^ 20 M microplate multimode reader. In this work, they have shown that this approach, can identify efficiently and quantify different types of cell death, including ferroptosis in tumor spheroids. 3D ELTA will enable the study of high-throughput cell death responses of tumor spheroids to therapy. Several issues arose during this study, including higher intensity of Sytox in stimulated cells compared to Triton X-100, resulting in >100% cell death; incomplete trypsinization of D10 spheroids; When plated, the spheroids disintegrated after 1 day during the ferroptosis assay; results were overestimated due to, for example, high intensity. Ways to solve the problem include more vigorous resuspension cells for 1 h; having an incubation step with collagenase I instead of trypsin; operating a suspension plate instead of an adherent plate to prevent cells from sticking to the plate; utilizing lower gains to optimize gain settings for new cell lines. The assay and 3D model results will be more clinically representative of the therapeutic response and improve the success rate of translating drugs into clinical trials.

## Discussion

The research on ferroptosis is in the ascendant, and many mechanisms are not yet precise. However, it can be considered a regulated cell death process mainly due to the damage to the intracellular redox protection system and the destruction of the membrane structure caused by the imbalance of iron homeostasis (Qu et al., 2022). Ferroptosis is a double-edged sword. On the one hand, it can eliminate pathological or infected cells to maintain the body’s homeostasis and continuity. On the other hand, it may damage normal cells and cause disease. Induction of exogenous stimuli and substances that promote or prevent ferroptosis play different roles in different pathological conditions (Chen et al., 2021; Koren and Fuchs 2021). As a unique programmed cell death process, ferroptosis has shown great potential and uniqueness in experimental and treatment-resistant cancer models. Therefore, the US Food and Drug Administration has approved octamine, sorafenib, and silica nanoparticles as ferroptosis inducers to treat tumors (Zhu et al., 2022).

The induction of ferroptosis in tumor cells is one of the emerging anti-tumor therapeutic strategies, and its related ferroptosis-inducing drugs have been applied in clinical tumor treatment. Although it has brought prospects for tumor patients, numerous problems still have not yet been solved. The research on tumor ferroptosis also requires to be carried out from the perspective of the intrinsic characteristics of tumors (Del Valle et al., 2020; Su et al., 2020). Various aspects of the tumor microenvironment have been involved in ferroptosis, such as inflammation, immunity, stromal cells, angiogenesis, and metabolism. As described above (Takahashi et al., 2020), they encountered that 2D culture conditions display little sensitivity to NRF2 knockdown, while the expansion of cells carrying NRF2-activating mutations is dramatically impaired in 3D culture. Under 3D spheroid growth conditions, spheroid cores deprived of extracellular matrix undergo higher reactive oxygen species (ROS), lipid peroxidation, ferroptosis, and central scavenging. NRF2 activation facilitates 3D growth by interfering with ferroptosis and increasing core survival. In the future, ferroptosis can be linked to the tumor microenvironment, and further study the effects of various factors in the tumor microenvironment on ferroptosis. These findings provide several insights into tumor therapy (H. Wang et al., 2021). At present, some factors affecting ferroptosis in the tumor microenvironment have been unearthed. Screening or synthesizing drugs with synergistic effects in combination with ferroptosis-inducing drugs by 3D culture methods is a prospective way to explore anti-tumor drugs. The synergistic ferroptosis medicines may improve the prognosis of tumor patients by accelerating the ferroptosis of tumor cells and increasing the efficacy of ferroptosis-inducing drugs in clinical anti-tumor therapy. The in-depth research on the effect of the tumor microenvironment on ferroptosis by the 3D culture method will open a new chapter in anti-tumor treatment.
